# A health promotion perspective on One Health

**DOI:** 10.17269/s41997-024-00872-y

**Published:** 2024-03-13

**Authors:** Evelyne de Leeuw, Ilona Kickbusch, Simon R. Rüegg

**Affiliations:** 1 ESPUM, École de santé publique de l’Université de Montréal, Montreal, QC Canada; 2 University of New South Wales, Sydney, Australia; 3 WHO European Regional Office Technical Advisory Group One Health, Copenhagen, Denmark; 4 Global Health Centre at the Graduate Institute in Geneva, Le Grand-Saconnex, Switzerland; 5 Vetsuisse Faculty, University of Zurich, Zurich, Switzerland

**Keywords:** One Health, Health promotion, Policy, Integration, Une seule santé, promotion de la santé, politiques, intégration

## Abstract

The One Health concept has acquired increasing attention due to the COVID-19 pandemic. We argue for a health promotion perspective that frames One Health in terms of positive health for people, animals, and ecosystems and includes a spiritual-cosmological dimension. This would enhance policy, research, and practice across disciplines and sectors for a more resilient and harmonious planet.

## Résumé

Le concept « Une seule santé » fait l’objet d’une attention accrue en raison de la pandémie de COVID-19. Nous plaidons en faveur d’une perspective de promotion de la santé qui définit Une seule santé en termes de santé positive pour les personnes, les animaux et les écosystèmes et qui inclut une dimension spirituelle et cosmologique. Cela permettrait d’améliorer les politiques, la recherche et la pratique dans toutes les disciplines et tous les secteurs afin d’assurer une planète plus résiliente et plus harmonieuse.

## Moving One Health closer to well-being

The concept of “One Health” has firmly entered local and global political and professional narratives. The COVID-19 pandemic has opened perspectives that allow a wider understanding of what health is. The complex interface of factors that determine health at many levels, from geosphere and biosphere to microbiological interactions, has acquired many labels—including planetary health, ecohealth, and others (Assmuth et al., 2019).

This is supported by a range of expert-driven developments (e.g., the One Health High-Level Expert Panel, OHHLEP et al., 2023), which challenge prevailing concepts of public health and global health. While these approaches recognize health as an emergence of complex interactions, they have not yet addressed the prevailing disease-centred perspectives in health governance. “One Health” has yet to embrace well-being agendas. We propose to go the next step towards a more meaningful and positive integration of the ever-growing awareness that human health and the balance of lands, airs, waters, ecosystems, plants, animals, people, and communities are intricately enmeshed (Gosh, 2021).

**Fig. 1:**
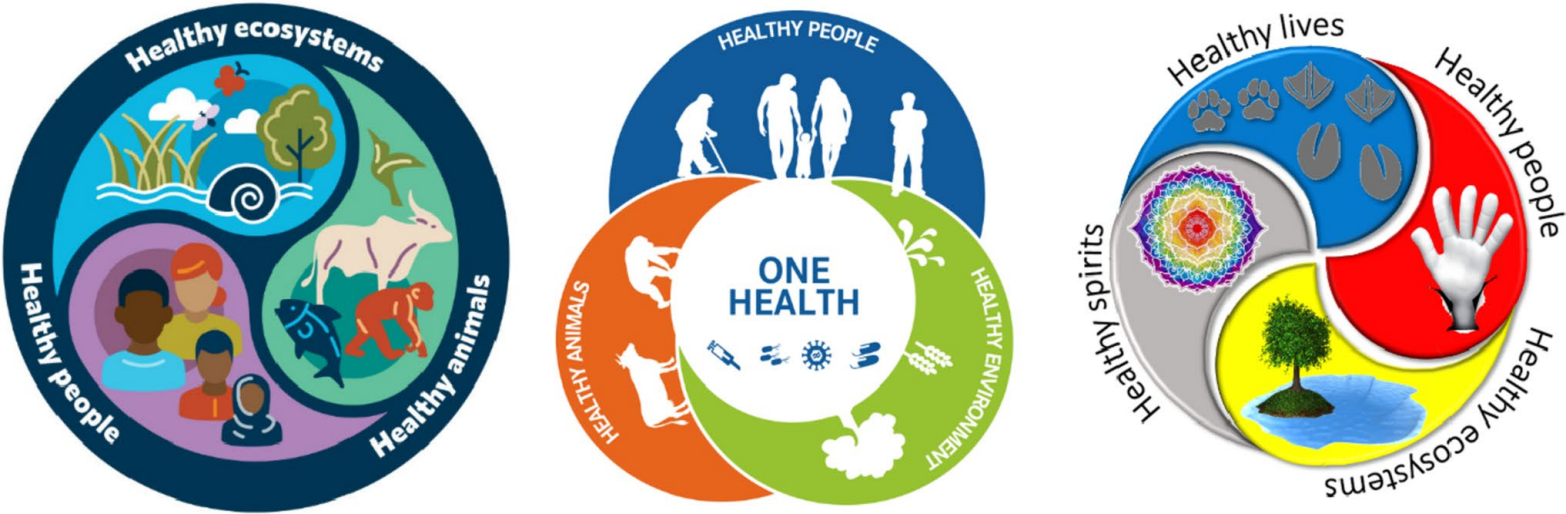
Three visual conceptualizations of a unified health promotion One Health perspective

From such a perspective, One Health conceptualizations and its practice can further evolve from very technical problem and disease focused framings towards an increasing salutogenic understanding of a health and well-being paradigm as expressed in the illustrations below (see Fig. 1). These show remarkable convergence with the Ottawa Charter for Health Promotion (de Leeuw & Harris-Roxas, 2016). However, the extension of health promotion beyond humans will need the full engagement and support of people and communities, also on behalf of the biosphere that sustains them.

## Recent One Health developments

The One Health perspective has been adopted by the Quadripartite (an alliance of four global organizations; FAO, UNEP, WHO, and WOAH, 2022) and taken forward in calls by a wide range of organizations to take action at all levels of governance (e.g., Rock et al., 2009). In Europe, the report of the “Monti Commission” urges for a unified vision of health (Monti et al., 2021). It has been proposed as a leading concept for pandemic preparedness, and proposed for inclusion in new international agreements, for example a pandemic accord (Kickbusch & Holzscheiter, 2021). Others have called for a more unified approach to the “oneness of one health” (e.g., de Leeuw, 2022; Laaser & Seifman, 2023). With notable exceptions—in, for instance, Quebec and Costa Rica—the mainstream conceptualizations of One Health prevailing at political and operational level are still patho-centric, give little consideration to animal well-being, undervalue the health benefits of nature, and neglect the spiritual dimension of health. Addressing these would allow us to develop a conceptual and operational understanding of health fit for the twenty-first century.

### Healthy ecosystems

In the prevailing discourse of One Health, the intrinsic value of nature is debated, but to satisfy the current, evidence-based policy processes, the value of nature is translated into ecosystem-services (to humans) which are then given an economic value (Costanza et al., 1997). However, building on a logic that healthy ecosystems provide human well-being via ecosystem services, a review of the Ecosystem Services Valuation Database found that approximately 58% of the records data on ecosystem health were lacking (Hernández-Blanco et al., 2022). Independently, the re-emergence of ecological perspectives in human and animal health originating from environmental psychology, human geography, and other disciplines, provides abundant evidence supporting the health benefits from nature rather than risks (Locke et al., 2021; Nishi & Hashimoto, 2022), and fuel the need to reconsider our relationship to nature (Zu Ermgassen et al., 2022).

### Healthy people

Yet, most of the current One Health discourse is still driven by virology, bacteriology, parasitology, pathology, and (veterinary and human) diagnostic and clinical perspectives. By focussing on a very narrow concept of causal pathways, this stance ignores the context of pathogenesis and links One Health very much to *disease* outbreaks and pandemics. This biomedical approach to public health which is reflected in many One Health concepts has hindered socially and ecologically relevant and responsive progress over the last five decades or so (Potvin & Jones, 2011). First, it adopts a deficit model to health (focusing on disease and dysfunction). Second, it wrongly assumes biological, spatial, and cultural homogeneity among human populations—exacerbating unacceptable inequities (e.g., Braam, 2022), showing the fragile balance between forced relocation, livelihoods, and livestock health and subsequent emerging and exacerbating inequities. One of us (Rüegg et al., 2017) has led exactly such efforts, albeit from an academic rather than community-driven positive health perspective.

### Healthy animals

Furthermore, veterinary public health still puts itself primarily at service to protect human health, with little consideration of animal well-being or health other than from an economic perspective (Pinillos et al., 2016). For example, stamping out (“culling” or brutally put, *killing*) whole populations to avoid disease transmission (among animals or from animals to humans) is recommended by the World Organisation for Animal Health under given circumstances. The discussion about animal rights is highly debated as it questions this utilitarian stance and would cause serious dilemmas in daily practice as a consequence of meat-based diets, or drug registration requirements. Nevertheless, there is an emerging recognition that animal well-being (indeed, *health promotion*) has an implicit value for itself and is tightly linked to human well-being. Also, there is increasing awareness that, e.g., in subsistence farming or pet ownership, the value of animals is not amenable to a purely economic analysis. Considering animals and their health and well-being from more than a biomedical or agro-economic perspective remains challenging and will require societal dialogue, including on the role of meat in our diet. There is more to a reduced meat consumption than just methane emissions or anthropocentric arguments. This is reflected in the “Nature Interaction Pattern” (Kahn, 2022) that conceptualizes a more meaningful engagement among animals, humans, and nature systematically.

### Healthy spirit

A perspective commonly missing from the current One Health discourse is that of belief systems, cosmologies, or spirituality. A strong body of literature is emerging (e.g., Hillier et al., 2021; McMillan et al., 2016) that situates institutional and governance perspectives around sovereignty and the one-ness of the planet in a strong, evidence-based One Health/Well-being agenda. It is clear that particular worldviews shape the balance among human, natural, and ecosystems connections. For instance, for many Indigenous peoples, humanity is indelibly part of the temporal, natural, and spiritual world. “Connection to Country” is health-making, or salutogenic (Thorpe et al., 2023). All creation is one, and it is impossible for many Indigenous peoples to conceive that one species or mob (tribe) can claim ownership of a particular spatial phenomenon over others. In fact, Maori people in Aotearoa successfully claimed legal personal rights to features in the natural environment such as rivers and mountains—such developments open up new challenges and opportunities for a united One Health view. For example, an inscription on the Indian parliament states Vasudhaiva Kutumbakam (*One World—One Family—One Future*) and inspired the newly established Global Centre for Traditional Medicine, GCTM (Patwardhan et al., 2023). Similar points are put forward by A. Gosh (2022) in describing world views of the Yanomami from the Amazon basin. When it comes to more anthropocentric views, in medical anthropology, a therapeutic unit was hypothesized to frame the Maya healing practices in Guatemala. This unit would extend the therapeutic relationship beyond the patient–healer dyad and include family, community, as well as the natural and spiritual realms into a coherent system required to achieve success (Berger-González et al., 2016). Similar holistic framings are reported from ethnoveterinary medicine in Africa (McCorkle & Mathias-Mundy, 1992). These ideas are powerful catalysts for a positive One Health development.

## One Health Promotion

Based on these notions, we echo Stephen’s (2020) call for a health promotion approach to One Health. Over the four years since this seminal work was published, the context has changed considerably. We consider this to be the time to position the current One Health developments squarely in two important perspectives: 1) a health promotion and salutogenic one; and 2) a focus on the spaces, places, and contexts where a healthy One Health plays out (de Leeuw, 2020, 2022).

The health promotion approach (also positive health, or asset-based approach) is a powerful coagulant for inter- and transdisciplinary thinking. This is forcefully demonstrated by the recent three volumes of the Global Handbook of Health Promotion Research, which firmly adopted a reflexive stance to inclusive knowledge generation and application—a notion well tuned to the needs for an emancipatory, diverse, and comprehensive view of ecological, planetary, and One Health. Such thinking will be critical for the success of One Health solutions of tomorrow (Whittaker et al., 2021; Kickbusch, 2021).

The current deliberations with(in) the One Health community recognize that the boundaries of the discourse need to move beyond more-than-human, inter-species and clinical emergency preparedness. Transdisciplinary action trajectories in, for instance, new networked governance approaches and joined-up government models have been successfully explored and validated in the health promotion field—in Healthy Cities, Healthy Schools, Healthy Islands, and other glocal network settings (Dooris et al., 2022). Essentially, the strong emphasis of One Health on prevention at the systemic level (Adisasmito et al., 2022) aligns with the idea of One Health Promotion or the promotion of a healthy “One”.

The health promotion literature abounds with conceptual and practical approaches to healthy people and healthy settings (e.g., Dooris et al., 2022)—including most recently the digital world (e.g., Lupton, 2022), and examples of how to practice joined-up governance and how to address inequity and empower communities. The wealth of this knowledge has so far not been made use of in the One Health nor the planetary health debate.

It is time to re-emphasize and reinvigorate the notion that health is not merely the absence of disease or infirmity, but a systemically inherent aspiration of the entire planet in all its wondrous diversity. With the renewed emphasis on One Health in the Quadripartite Plan of Action comes an opportunity to invest in health of nature, ecology, spirit, and balance, to the benefit of all. Including that crust of humanity. A One Health Promotion perspective would generate stability, harmony, and resilience—it would make our world an ever better one.

## Data Availability

Not applicable.
